# Tsetse Flies Infected with Trypanosomes in Three Active Human African Trypanosomiasis Foci of the Republic of Congo

**DOI:** 10.3390/pathogens11111275

**Published:** 2022-10-31

**Authors:** Irina Bemba, Arsene Lenga, Herman Parfait Awono-Ambene, Christophe Antonio-Nkondjio

**Affiliations:** 1 Laboratory of Animal Biology and Ecology, Faculty of Science and Technology, Marien Ngouabi University, Brazzaville B.P. 69, Congo; 2 Institut de Recherche de Yaoundé (IRY), Organisation de Coordination pour la lutte Contre les Endémies en Afrique Centrale (OCEAC), Yaoundé B.P. 288, Cameroon

**Keywords:** transmission, tsetse flies, HAT, Republic of Congo

## Abstract

Introduction: Human African trypanosomiasis (HAT) is a neglected tropical disease still endemic in the Republic of Congo. Despite the continuous detection of HAT cases in the country, there is still not enough data on trypanosome infections in tsetse flies, trypanosome species and tsetse flies’ species distribution in endemic foci. The present study was intended to fill this gap and improve understanding of trypanosome circulation in three active foci in the centre and south of Congo. Methods: Pyramid traps were set in various places in villages to collect tsetse flies both during the rainy and dry seasons. Once collected, tsetse flies were identified using morphological keys. DNA extracted from flies was processed by PCR for species identification and for detection of trypanosome presence. A second PCR was run for different trypanosome species identification. Results: A total of 1291 tsetse flies were collected. The average apparent density of flies per day was 0.043 in Mpouya, 0.73 in Ngabé and 2.79 in Loudima. *Glossina fuscipes quazensis* was the predominant tsetse fly collected in Ngabé and Mpouya, while *Glossina palpalis palpalis* was the only tsetse fly found in Loudima. A total of 224 (17.7%) flies were detected infected by trypanosomes; 100 (7.91%) by *Trypanosoma congolense savannah*, 22 (1.74%) by *Trypanosoma congolense forest*, 15 (1.19%) by *Trypanosoma vivax*, 83 (6.56%) by *Trypanosoma brucei* (s.l.) and 2 (0.16%) undetermined species. No T *Trypanosoma brucei gambiense* was found. A total of 57 co-infections between *T. brucei* (s.l.) and *T. congolense savannah* or *T. brucei* (s.l.) and *T. congolense forest* were found only in *G. p. palpalis*. Loudima recorded the highest number of infected tsetse flies. Conclusion: The study provided updated information on the distribution of tsetse fly populations as well as on *Trypanosoma* species circulating in tsetse flies in the different active HAT foci in Congo. These data suggested a high risk of potential transmission of animal trypanosomes in these foci, thus stressing the need for active surveillance in this endemic area.

## 1. Introduction

African trypanosomiases are parasitic diseases affecting humans and animals, caused by many species of trypanosomes and transmitted by tsetse flies of the genus *Glossina* [1]. Many species of trypanosomes infect cattle, including *T. brucei, T. congolense* and *T. vivax* [2]. These species cause African animal trypanosomiasis (AAT), resulting in livestock losses and reduced productivity. *T. brucei rhodesiense* and *T. brucei gambiense* are known to infect humans, causing human African trypanosomiasis (HAT), which is a fatal disease. *T. b. gambiense* is common in central and west Africa, while *T. brucei rhodesiense* is common in east and south Africa. They are known to cause chronic and acute forms of the disease, respectively [3,4,5].

HAT is one of the neglected tropical diseases that has been targeted by WHO for elimination as a public health problem by 2030. A steady decrease in the disease has been reported since 2014. In 2020, a total of 565 gambiense-HAT (g-HAT) cases and 98 rhodesiense-HAT (r-HAT) cases were reported [6]. At the national level, HAT elimination as a public health problem requires an average of less than 1 case per 10,000 inhabitants as registered for at least five years in affected health districts or countries [6]. Recently five countries, including Cote d’Ivoire, Togo, Benin, Uganda and Equatorial Guinea, were declared free of HAT [7]. Only twenty countries across sub-Saharan Africa are still affected, and about 55 million people are at risk of HAT transmission [6]. However, the disease is heterogeneously distributed across the continent, with the Democratic Republic of Congo registering close to 70% of total cases [6]. The main goal for 2030 is the elimination of transmission [6]. Rural populations engaged in farming, fishing, herding or hunting have an increased risk of being bitten by tsetse flies, with *T. brucei gambiense* as the most prevalent species, which accounts for 87% of reported sleeping sickness cases [6].

Despite a reduction in the number of cases, in the Republic of Congo, the disease remains endemic. Between 2014 and 2017, cross-sectional surveys conducted in different active foci in the country, notably in the Bouenza, Plateau, Pool and Cuvette regions, reported that 90 infected people out of 36,725 people were examined [8]. It was estimated that a total of 50 g-HAT cases were recorded in the country between 2019 and 2021 [6,9]. Only g-HAT is present in the country. Despite efforts made by the government and partners, sleeping sickness persists in many foci. To better understand the epidemiology of human and animal trypanosomiasis, it is important to generate data on the distribution and dynamics of tsetse fly populations [3,10]. To date, there is a lack of data on the distribution and population dynamics of tsetse flies and their trypanosome parasites in active HAT foci in the Republic of Congo. The latest entomological studies on HAT in Congo date back to the 1990s; as a result, the description of the epidemiology of HAT in the Republic of Congo is poor and out-of-date. The National Control Programme for sleeping sickness (PNLTHA) in Congo, due to a lack of resources, mainly limits its activities to diagnostic campaigns and the treatment of the population at risk.

The aim of the present study was to generate entomological data on tsetse fly species caught in three endemic foci in the Republic of Congo and parasitological data about their trypanosome infections.

## 2. Materials and Methods

### 2.1. Study Sites

The study was conducted in three active foci situated in the south and centre of the Republic of Congo.

**Figure 1:**
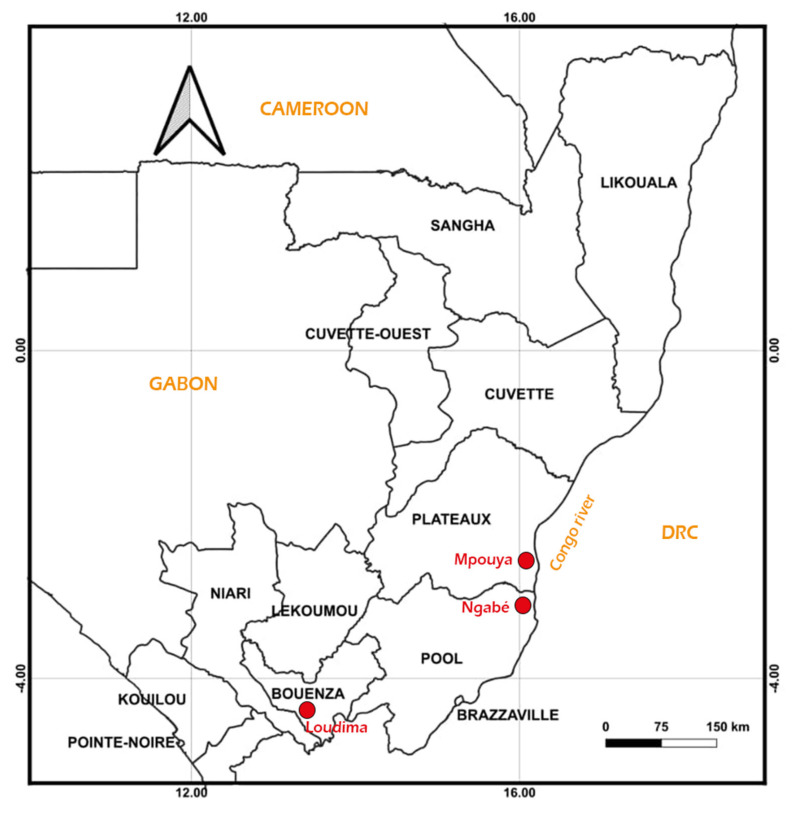
Map of the Congo Republic showing the situation of study sites. (Source: map made withQGIS V. 3.22 (Sutton T, Dassau 0, Sutton M, Nsibande L, Mthombeni S. 2022 QGIS Desktop User Guide/Manual (QGIS 3.22) Białowieża, Poland)).

− Mpouya (2°36′57″ S, 16°12′43″ E) is located in the Plateau administrative division about 300 km north of Brazzaville. The focus is composed of several villages bordering the Congo River. Its vegetation is essentially a savannah with a few tall trees, and the population lives on fishing and farming.
− Ngabé (3°12′52″ S, 16°10′1″ E) is situated about 200 km north of Brazzaville in the Pool region. This focus is also composed of several villages bordering the Congo River. The vegetation is savannah and forest galleries along the edges of rivers. The population mainly practices farming, hunting and fishing for a living.
− Loudima (4°6′45″ S, 13°3′30″ E) is located in the Bouenza division about 300 km south of Brazzaville. The vegetation is dominated by grass fields, and the main economic activity of the population is farming (Figure 1).

Tsetse flies sampling was conducted in four to five villages in each of these foci.

### 2.2. Tsetse Flies Sampling

Entomological surveys were conducted in November 2019, November 2020 (rainy seasons) and June 2021 (dry season). During each survey, pyramid traps [11] were placed in different places (close to the river or swamps, in shaded areas and in the villages) for at least three days in five villages in Ngabé and four villages in Mpouya and Loudima. A total of 251 traps were set, including 106 in November 2019, 94 in November 2020 and 51 in June 2021. For each trap, the geographical coordinates were recorded using a GARMIN GPS (eTrex^®^ 10). The minimum distance between traps was 100 m. The traps were visited twice daily (10 A.M. and 4 P.M.). Once the tsetse flies were captured, they were morphologically identified to determine the sex and species [12] and then preserved in microtubes containing alcohol. In the field, these microtubes were kept in a refrigerated cooler (at +4 °C) and in the laboratory at −20 °C until DNA extraction.

### 2.3. DNA Extraction

DNA was extracted from the whole tsetse fly by the cethyl trimethyl ammonium bromide (CTAB) method [13]. In the laboratory, the alcohol was evaporated at room temperature, and 600 µL of CTAB buffer (5% CTAB; 1 M Tris, pH 8; 0.5 M EDTA, pH 8; 5 M NaCl) was added. Then tsetse fly was ground with a pestle and then incubated in a water bath at 60 °C for 30 min. Then 600 µL of chloroform/isoamyl alcohol (24/1) was added to each tube. This mixture was slowly homogenised for 15 min and then centrifuged at 13,000 rpm for 15 min. The upper aqueous phase was removed and transferred to another 1.5 mL microtube. The DNA was precipitated by the addition of 600 µL isopropanol. After gentle homogenisation of each microtube for 5 min and incubation overnight at −20 °C, each microtube was centrifuged at 13,000 rpm for 15 min. The DNA pellet was then washed twice with cold 70% ethanol and dried overnight at room temperature. The resulting DNA pellet was resuspended in 50 µL of sterile nuclease-free water and stored at −20 °C or used directly for PCR.

### 2.4. Molecular Identification of Tsetse Fly Species

The principle of this PCR was based on the amplification of the ITS1 region (“internal transcribed spacer 1”) of the tsetse genome, using the primer pair “Diag forward” (5′-TGGACTTCGGATTAAGTACAACA-3′) and “Diag reverse” (5′-TCATTATGCGCTATTAAGGTAAGC-3′) [14]. For this identification, only one PCR cycle was performed in a final volume of 25 µL containing 1× PCR buffer (10 mM Tris-HCl (pH 9.0), 50 mM KCl), 3 mM MgCl_2_, 1 µL (15 pmol) of each primer, 0.5 μL (200 mM) of dNTPs, 0.1 μL (one unit) of Taq DNA polymerase, 2.5 µL of DNA and 17.4 μL of sterile water. The amplification programme began with a denaturation step at 95 °C for 3 min and 30 s, followed by 30 cycles of amplification; each cycle contained a denaturation step at 95 °C for 30 s, a hybridisation step at 56 °C for 1 min and an extension step at 72 °C for 1 min, followed by a final extension step at 72 °C for 5 min.

The amplification products were run on a 2% agarose gel to enable species identification.

### 2.5. Molecular Identification of Different Trypanosome Species

The identification of trypanosomes was performed by the amplification of the internal transcribed spacer 1 (ITS1) of the ribosomal DNA of different trypanosome species, as described by Ravel et al. [15]. For this identification, two PCR cycles were performed; the first cycle was performed in a final volume of 25 µL containing 1× PCR buffer (10 mM Tris-HCl (pH 9. 0), 50 mM KCl), 2 mM MgCl_2_, 1 µL (10 pmol) of each primer (5′-CAA ATT GCC CAA TGT CG-3′ and 5′-GCT GCG TTC TTC AAC GAA-3′), 0.5 µL (200 mM) of dNTPs, 1 µL (one unit) of Taq DNA polymerase (5 U/µL), 5 μL of DNA and 14 µL of nuclease-free water. The amplification program began with a denaturation step at 94 °C for 3 min and 30 s, followed by 30 cycles of amplification; each cycle contained a denaturation step at 94 °C for 30 s, a hybridisation step at 58 °C for 1 min and an extension step at 72 °C for 1 min, followed by a final extension step at 72 °C for 5 min.

The amplified products from the first round of PCR were diluted 10-fold, and 3 µL of each dilution was used as the template for this round. The second round of PCR was performed with two different primers (5′-CCT GCA GCT GGA TCA T-3′ and 5′-ATC GCG ACA CGT TGT G-3′), which amplify a specific area of the ITS1 fragment previously amplified. The amplification program was identical to that of the first round of PCR. After nested PCR, the amplicons were separated by electrophoresis on a 2% agarose gel which was then stained with Midori green advance DNA Stain (NIPPON Genetics EUROPE GmbH) and visualised under UV light.

Trypanosome species were identified based on the length of ITS1 fragments. The fragment size was about 650 bp for *T. congolense* (630 bp for *T. congolense forest* and 610 bp for *T. congolense savannah*). For *T. vivax,* the expected fragment length was 150 bp, whereas it was 400 bp for trypanosomes belonging to the Trypanozoon subgenus (*T. brucei* (s.l.), *T. evansi* and *T. equiperdum*).

### 2.6. Identification of Trypanosoma Congolense Forest and Trypanosoma Congolense Savannah

After amplification of the ITS1 sequences, all samples that had a DNA fragment between 600–650 bp, corresponding to the expected size of *T. congolense*, were subjected to a further PCR where specific primers were used to separate *T. congolense forest* from *T. congolense savannah*. These specific identifications were performed as described by Simo et al. [16] using primers TCF1 (5′-GGA CAC ACG CCA GAA GGT ACT T-3′) and TCF2 (5′-GTT CTC TCG CAC CAA ATC CAA C-3′) for *T. congolense forest* “type” [17], and TCS1 (5′-CGA GCG AGA ACG GGC AC-3′) and TCS2 (5′-GGG ACA AAC AAA TCC CGC-3′) for *T. congolense savannah* “type” [18]. PCR reactions were performed in a final volume of 25 μL containing 1× PCR buffer (10 mM Tris-HCl (pH 9.0), 50 mM KCl), 3 mM MgCl_2_, 1 µL (15 pmol) of each primer, 0.5 μL (200 mM) of dNTPs, 1 μL (one unit) of Taq DNA polymerase, 3 µL of DNA and 16 μL of sterile water. The amplification program consisted of a denaturation step at 94 °C for 3 min 30 s, followed by 40 cycles of amplification, including a denaturation step at 94 °C for 30 s, a hybridisation step at 60 °C for 1 min and an elongation step at 72 °C for 1 min, followed by a final elongation step at 72 °C for 5 min.

The amplified products were separated by electrophoresis on a 2% agarose gel containing Midori green advance DNA Stain (NIPPON Genetics EUROPE GmbH). The DNA bands were visualised under ultraviolet (UV) light, and a picture of the gel was taken and stored.

### 2.7. Data Analysis

The apparent density of tsetse flies was calculated using the following formula: ADT = C/(T × D), where C represents the number of flies collected, T is the number of traps used and D is the number of days of trapping. The ANOVA was used to compare the ADT between foci. Statistical analyses were conducted using the software MedCalc [19]. The infection rate of tsetse flies between sites was compared using the Pearson chi-square test. The threshold for significance was *p* < 0.05.

## 3. Results

### 3.1. Entomological Surveys

During the three entomological surveys, 251 pyramid traps were set up and captured 1291 tsetse flies (190 in 2019, 462 in 2020 and 639 in 2021) (Table 1). The tsetse flies collected belonged to three species, including *Glossina palpalis palpalis* (*G. p. palpalis*), *Glossina fusca congolensis* (*G. f. congolensis*) and *Glossina fuscipes quazensis* (*G. f. quazensis*). *G. f. quazensis* was the most abundant fly in Ngabé and Mpouya, while *G. p. palpalis* was the only tsetse fly found in Loudima (Table 2). The highest ADT was recorded at the site of Loudima, whereas the lowest was recorded in Mpouya. A significant difference was observed when comparing the ADT between sites (*p* ˂ 0.05).

### 3.2. Molecular Identification of Trypanosomes

From the 1291 tsetse flies captured, 1265 were screened, and molecular examination revealed that 224 (17.7%) were infected by at least one trypanosome species. Different species of trypanosomes were detected: T. c. savannah, T. c. forest, T. vivax and T. brucei s.l. The main trypanosome species found in tsetse flies were T. brucei s.l. and T. c. savannah. Loudima presented the highest number of infected flies, followed by Ngabé. Some flies (N = 57) were detected with co-infections T.c.savannah + T. brucei and T.c. forest + T. brucei.

Table 3 shows that all co-infections were detected in Loudima. No fly was detected as infected by T. brucei gambiaense or T. brucei rhodesiense after further molecular analysis.

## 4. Discussion

Although active detection of sleeping sickness cases is almost regularly conducted in sleeping sickness foci in the Republic of Congo [8,20], updated data on tsetse flies distribution and trypanosome species circulating in the country is not available for the majority of foci. The present study was conducted to fill this gap in order to better understand the epidemiological situation of HAT in different active foci in Congo. Tsetse flies were more abundant in Loudima compared to Ngabé and Mpouya. The only species caught in Loudima was G. p. palpalis, while the most abundant species found in Ngabé and Mpouya was G. f. quazensis. Both tsetse flies are riverine and share similar ecological preferences [12]. Even though the distribution range of these species is similar to previous findings [21], slight changes in species abundance and composition were noted in our work compared with previous findings [22,23] and support the possible influence of environmental or anthropic changes (deforestation, the extension of human settlements and increase in agricultural land surfaces) on tsetse fly distribution as reported elsewhere [16,24]. In Ngabé, Loudima and Mpouya, in addition to the rapid growth of the population, which has multiplied by four since the 1990s [25], deforestation and the expansion of the agricultural frontier for the cultivation of groundnuts, cassava and maize may have reduced the suitable habitats for the tsetse flies. Due to the limited number of villages sampled and the short duration of field trips, it is possible that the study did not capture the full distribution range of tsetse flies in the area. Regular xeno-surveillance studies in both the forest and savannah foci for periods of 10 to 15 days per season are needed to capture the real distribution range of tsetse species in Congo.

Tsetse flies were more abundant during the dry season as compared with the rainy season. Frequent overflowing of rivers and the inundation of large areas during the rainy season could have killed larvae developing in the soil and limited the emergence of flies from larvae into the adult stage. River banks, swamps or places with high humidity have always constituted ideal breeding grounds for tsetse larvae [26].

Female flies were always more abundant than males and represented 60.42% and 58.27% of G. f. quazensis and G. p. palpalis, respectively. These findings are similar to data recorded by Gouteux et al. [27]. The sampling of both male and female flies in collection places suggests that these areas are both suitable for breeding and hunting for the flies.

G. f. quazensis and G. p. palpalis were detected as infected by trypanosomes. These species are major vectors of trypanosomiasis in riverine habitats [16,28,29]. Five trypanosome species, including: *T. congolense savannah*, *T. congolense forest*, *T. vivax*, *T. brucei* (s.l.) and an unknown species, were detected. The majority of trypanosome species recorded are responsible for African animal trypanosomiasis (AAT) or nagana. The frequent circulation of trypanosomes infecting animals is probably a serious threat to livestock in these foci. The proportion of tsetse flies infected by trypanosome was high and similar to findings recorded elsewhere [16,30]. Due to frequent records of sleeping sickness cases in humans, the transmission of trypanosomes infecting humans may also be occurring in the area. A high number *Trypanosoma brucei* s.l. infections were recorded, yet the molecular examination of these samples did not detect the presence of *T. b. gambiaense*. The fact that we could not detect any flies infected with *T. b. gambiense* could result from the low sample size of flies collected. Because of the very low number of cases currently recorded per year in Congo, the number of infected flies should be very low. Detecting infected flies will require screening a minimum of 4000 flies or more. Future xeno-monitoring studies should consider increasing the sampling effort. The prevalence of *T. congolense* savannah infections found in our work is in agreement with data published elsewhere [29,31,32].

Over 17.7% of flies collected were infected by at least one trypanosome that causes animal trypanosomiasis. It is possible that the true infection rates could have been overestimated due to the fact that the whole fly was processed by PCR to detect infections. Studies conducted so far indicated different infection rates when screening the midgut, the proboscis, the salivary glands or the whole body [16,33]. Moreover, since trypanosome DNA was used as proof of active infection, the infection rate may be overestimated because the DNA of dead trypanosomes could have been amplified [34]. The fact that less than 8% of the analysed flies had signs of having taken a blood meal significantly reduced the bias associated with the presence of trypanosomes in the blood, suggesting that the trypanosomes were already present inside the fly. It is also likely that both mature and immature infections were detected in our work because no dissection was conducted. Finally, the uncommonly high infection rate detected during this study requests further verification through large-scale passive and active screening of animals, humans, and tsetse fly populations.

Female tsetse flies were found to be more infected compared to males. This difference could result from the fact that females live longer than males [4,35]. Similar findings have been registered in Chad [33].

Mixed infections were detected during this study; this pattern is in line with studies conducted so far across Africa [31,36]. The presence of mixed infections could be associated with a high prevalence of these pathogens in mammalian hosts and probably frequent contact between tsetse flies and infected animals. Yet the presence of mixed infections was found to vary according to focus, time period or country [29,31]; factors influencing trypanosome abundance and diversity in a given focus are still not well understood and deserve further investigations.

Human African trypanosomiasis is still a public health concern in many countries in sub-Saharan Africa, and the emergence and spread of the disease could impede development efforts. In this light, it is important that immediate action be taken to achieve elimination goals the fastest possible [37]. Studies on tsetse fly distribution and trypanosome species circulating in both animals and humans are key to understanding the epidemiological situation of animal and human African trypanosomiasis and for the implementation of efficient control strategies aimed at eliminating sleeping sickness in endemic foci. The circulation of animal trypanosomes could have a damaging effect on the economy. By affecting animal health, trypanosomes, particularly T. congolense, reduce livestock production by affecting the quality and quantity of meat and limiting cattle-rearing activities [38]. Because anthropisation processes, such as deforestation, dam construction or the practices of intensive agriculture, have reduced the tsetse fly’s distribution range, the mapping of affected areas could be a key component of vector control activities on the field [39]. Targeted control activities could lead to the reduction of control costs and also improve the efficacy of control actions on the field [40].

In active foci, the use of insecticide-impregnated traps or screens could be promoted for improved control. The regular treatment of infected livestock with trypanocides could also be undertaken alongside these measures.

As possible limits of the study, it is likely that with less than 1300 flies collected during the present study, chances of bias, missing certain species or not being able to detect flies infected by g-HAT were high. Future studies should put more emphasis on increasing the sample size and geographically expanding the collection sites to capture species diversity and distribution range and to increase the duration of collection periods in order to capture the dynamic of circulation of trypanosome species.

## 5. Conclusions

The study provided updated information on the risk of human and animal trypanosomiasis in three active endemic foci in Congo almost 30 years after the last xeno-monitoring studies in Congo. A high circulation of animal trypanosomes was recorded in this area. It is likely that trypanosome infecting humans such as *T. b. gambiense* could be present since regular sleeping sickness cases are reported in these foci. These findings call for continuous surveillance using both xeno-surveillance and passive and active detection of cases in order to stop the dissemination of HAT cases to unaffected areas. HAT in Congo has significantly decreased due to concerted efforts, and this momentum needs to be maintained in order to achieve elimination goals by 2030.

## Figures and Tables

**Table 1:** Tsetse fly densities per study site.

Foci	No. Tsetse fly	No of Teneral Flies (%)	No. Trap	ADT	No of Males (%)	No of Females (%)	Sex Ratio
Mpouya	5	0 (0)	38	0.043	0 (0)	5 (100)	0
Ngabé	179	5 (0.03)	81	0.73	58 (32.40)	121 (67.60)	0.5
Loudima	1107	11 (0.01)	132	2.79	462 (41.73)	645 (58.27)	0.7
*p*-value				0.001			

No: number; ADT: apparent density of tsetse flies per trap per day.

**Table 2:** Distribution of different tsetse species from the three sleeping sickness foci of the Republic of Congo.

HAT Foci	Tsetse Fly Species			Total
	*G.p. palpalis*	*G.f. quazensis*	*G.f. congolensis*	
Mpouya	−	5	−	5
Ngabé	−	178	1	179
Loudima	1107	−	−	1107
Total	1107	183	1	1291

G.p. palpalis: Glossina palpalis palpalis; G.f. quazensis: Glossina fuscipes quazensis; G.f. congolensis: Glossina fusca congolensis.

**Table 3:** Molecular identification of trypanosomes in tsetse flies.

Foci	No. Tested	*T. c. s.* (%)	*T. c. f. (%)*	*T. vivax (%)*	*T. b.* (s.l.) (%)	*Coinfection T.b + T.c.s*	*Coinfection T.b + T.c.f.*	Unknown Species
Loudima	1103	95 (8.61)	21 (1.9)	11 (1)	82 (7.43)	49 (85.96)	8 (14.04)	4 (0.36)
Mpouya	5	0	0	0	1 (20)	0	0	0
Ngabé	157	5 (3.18)	1 (0.64)	4 (2.55)	0	0	0	0
Total	1265	100 (7.91)	22 (1.74)	15 (1.19)	83 (6.56)	49 (3.87)	8 (0.63)	4 (0.16)

No: number; T.c.s.: Trypanosoma congolense savannah; T.c.f.: Trypanosoma congolense forest; T.b (s.l.): Trypanosoma brucei (senso lato).

## Data Availability

The datasets supporting the findings of this paper are all included in this paper.
